# Evaluation of the intra‐ and interfractional tumor motion and variability by fiducial‐based real‐time tracking in liver stereotactic body radiation therapy

**DOI:** 10.1002/acm2.12292

**Published:** 2018-02-28

**Authors:** Zhiwen Liang, Hongyuan Liu, Jun Xue, Bin Hu, Bin Zhu, Qin Li, Sheng Zhang, Gang Wu

**Affiliations:** ^1^ Cancer Center Union Hospital Tongji Medical College Huazhong University of Science and Technology Wuhan China

**Keywords:** baseline shift, intrafraction amplitude variability, liver, stereotactic body radiotherapy, tumor motion

## Abstract

**Purpose:**

Tumor motion amplitude varies during treatment. The purpose of the study was to evaluate the intra‐ and interfraction tumor motion and variability in patients with liver cancer treated with fiducial‐based real‐time tracking stereotactic body radiotherapy (SBRT).

**Methods:**

Fourteen liver patients were treated with SBRT using a CyberKnife. Two to four fiducial markers implanted near the tumor were used for real‐time monitoring using the Synchrony system. The tumor motion information during treatment was extracted from the log files recorded by the Synchrony system. Logfile‐based amplitudes in the superior–posterior (SI), left–right (LR) and anterior–posterior (AP) directions were compared to the 4DCT‐based amplitudes. The intra‐ and interfraction amplitude variations and the incidence of baseline shifts were analyzed for 66 fractions administered to 14 patients.

**Results:**

The median (range) logfile‐based liver motion amplitudes for all patients were 11.9 (5.1–17.3) mm, 1.3 (0.4–4) mm and 3.8 (0.9–7.7) mm in the SI, LR and AP directions, respectively. Compared with the logfile‐based amplitude, the 4DCT‐based amplitude was underestimated (*P* < 0.05). The median (range) intra‐ and interfraction liver motion amplitude variations were 4.3 (1.6–6.0) mm (SI), 0.5 (0.2–2.2) mm(LR) and 1.5 (0.3–3.3) mm (AP) and 1.7 (0.5–4.6) mm (SI), 0.3 (0.1–3.0) mm (LR) and 0.7 (0.3–2.7) mm (AP), respectively. Baseline shifts exceeding 2 mm, 3 mm and 5 mm were observed in 27.3%, 7.6% and 3% of the measurements, respectively, within 10 min, and in 66.7%, 38.1% and 19%, respectively, within 30 min for the square root of the sum of the squares of the distances in the SI, LR and AP directions (3D). The tumor motion amplitude was found to be correlated with the baseline shift.

**Conclusions:**

Most patients showed significant intra‐ and interfraction liver motion amplitude variations over the entire course of radiation. More caution is needed for patients with large tumor motion amplitudes.

## 1 INTRODUCTION

Radiation‐induced liver disease (RILD) has the potential to lead to liver failure, and the risk of RILD is correlated with the mean dose to the liver.^1^ With the use of stereotactic body radiation therapy (SBRT) for the treatment of liver tumors, it became feasible to achieve high rates of tumor control while minimizing the irradiation of the surrounding uninvolved liver, thereby reducing the risk of RILD.^2^, ^3^ SBRT needs to precisely deliver a highly conformal dose to the target in fewer fractions. Hence, tumor motion evaluation and its use in treatment planning are important for liver SBRT.

There are several methods for determining liver tumor motion in a simulation process, including four‐dimensional computed tomography (4DCT),^4^ inhale/exhale breath‐hold CT and cine magnetic resonance imaging (MRI).^5^ Due to the density difference between a tumor and the surrounding normal tissue, liver tumors are generally only visible in contrast‐enhanced CT scans. A synchronized contrast injection method during 4DCT simulation has been reported to account for the problem.^4^, ^6^ However, because intravenous contrast is not routinely used during pretreatment, it is difficult to recognize the tumor in cone beam CT (CBCT) images, and accurate patient alignment becomes a challenge in liver SBRT. Fiducials implanted near the liver tumor have been shown to be effective surrogates and are highly recommended.^2^ Bertholet et al. compared four CBCT‐based setup strategies for liver tumor. They concluded that marker‐based setup was substantially better than bony‐anatomy setup.^7^

Tumor motion patterns may change during the treatment, with either intra‐ or interfraction changes. Case et al. investigated intra‐ and interfractional tumor motion variability using CBCT^8^ or respiratory‐correlated CBCT.^9^ However, CBCT or respiratory‐correlated CBCT only represents the tumor motion over multiple respiratory cycles during image acquisition. Real‐time data from the treatment can provide information with more detail about the tumor motion. Using real‐time data, Malinowski et al.^10^ showed that the tumor baseline position can change in most treatment fractions in lung and pancreatic cancer patients.

Understanding tumor motion before and during treatment is important for high‐quality SBRT treatments, especially for those in which real‐time tumor tracking is not available. For this purpose, we investigated the logfile‐based tumor motion amplitude, intra‐ and interfraction tumor motion variability and baseline shift during the course of treatment. The results might contribute to current knowledge regarding liver tumor motion management.

## 2 MATERIALS AND METHODS

### 2.A Patients

In our institution, liver SBRT patients were treated with a robotic CyberKnife (Accuray Incorporated, Sunnyvale, USA) using the Synchrony Respiratory Tracking System (Accuracy Incorporated, Sunnyvale, USA, Versions 10.05.x). The Synchrony system recorded the real‐time tumor motion data and other information in logfiles. Between April 2017 and October 2017, 14 consecutive liver treatment patients with a total of 66 fractions were included in the study. Patients were treated with 3–10 fractions, for a total dose ranging from 40 to 50 Gy. The median (range) treatment time per fraction was 39 (33–49) min. The patient characteristics are reported in Table 1.

**Table 1:** Patient characteristics (*N* = 14)

Characteristics	
Age: Median (range) (yr)	57.5 (31–72)
Disease:	
Primary liver cancer	3 (21%)
Liver metastasis	11 (79%)
GTV Volume: Median (range) (cc)	3.1 (0.3–19.1)
Total dose: Median (range) (Gy)	46.5 (40–50)
Dose per fx: Median (range) (Gy)	9.5 (5–14)
Fraction: Median (range)	5 (3–10)
Treatment time per fraction: Median (range) (min)	39 (33–49)

### 2.B Simulation, prescription and treatment planning

All patients were implanted with 2–4 fiducials near the tumor under CT guidance.^11^ Simulation was typically administered 7–10 days after the implantation, which can minimize the risk of marker migration. The patients were supine with their arms along their sides and were immobilized with a customized vacuum body mold. Exhalation breath‐hold contrast‐enhanced planning CT was used for treatment planning. An additional 4DCT was used to evaluate tumor motion in all patients in case dynamic tracking was not possible during treatment.

The gross target volume (GTV) was expanded by a 5‐mm radial and 8‐mm craniocaudal margin for the planning target volume (PTV). The radiation dose was prescribed according to the isodose surface covering the PTV, typically 75% to 85% of the maximum PTV dose. SBRT was planned and delivered using the CyberKnife under free breathing.

### 2.C Synchrony respiratory tracking system

The primary concept of the tracking treatment using the Synchrony system is to build a correlation model between the internal tumor location and the external marker position.^12^ The internal tumor location was determined using the centroid of fiducials, which were implanted near the tumor before simulation, and the external optical marker was attached to a vest worn by the patient during the treatment. The correlation model is updated with each new X ray acquisition (typically every 60 s), thus accounting for the change of motion pattern during treatment. More detailed information can be found in the reference manual.^13^

The accuracy of the correlation model has been evaluated in a previous publication.^14^ The mean correlation model errors were less than 0.3 mm in their study, indicating that use of the correlation model to predict the tumor position is reliable. The internal tumor position throughout the treatment was estimated using the correlation model and was stored in log files (Modeler.log).

### 2.D Tumor motion analysis

Tumor motion amplitudes, baseline drifts and intra‐ and interfraction amplitude variability during treatment were evaluated.

**Figure 1:**
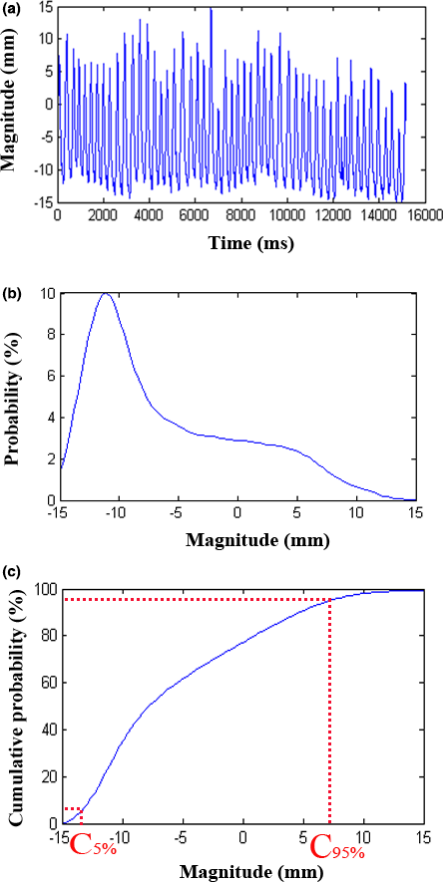
A schematic diagram for the definition of logfile‐based tumor motion amplitudes. The breathing cycles (a), the corresponding tumor position probability distributions (b) and the cumulative probability as a function with the tumor position (c). C5% and C95% are the cutoff points.

**Logfile‐based tumor motion amplitudes** were defined as the range of a 5% cutoff in each of the deepest inhale and exhale positions. To determine the 5% cutoffs, the probability distribution functions (PDFs) of tumor positions were calculated using the data recorded in the logfiles [Fig. 1(b)]. The cumulative probability as a function of tumor positions also be calculated: the corresponding positions of 5% (C_5%_) and 95% (C_95%_) cumulative probability were the cutoffs [Fig. 1(c)], and the logfile‐based amplitude was the range between C_5%_–C_95%_. This logfile‐based amplitude was compared with the amplitudes decided by the 4DCT.

**The intrafraction amplitude variability** was defined as the standard deviation (SD) of the peak‐to‐peak distance (the peak‐to‐peak distance was defined as the distance of each inhale peak to the next exhale peak) during one treatment fraction.

**The interfraction amplitude variability** was defined as the SD of the logfile‐based tumor motion amplitude over the entire treatment course.

**Baseline shift** was defined as a change in the mean tumor position from the baseline position across 10‐min blocks of data, The mean tumor position of the first 5 min after starting the treatment was taken as the baseline tumor position. The incidence of baseline shift exceeding 2 mm and 5 mm were evaluated, based on the consideration of (a) a > 2‐mm setup error, which required realignment for those patients treated with conventional LINAC at our institution; and (b) a 5–8 mm GTV to PTV margin. For comparison with the other publication,^15^ the incidence of baseline shift exceeding 3 mm was also calculated.

## 3 RESULTS

### 3.A Tumor motion amplitude

**Figure 2:**
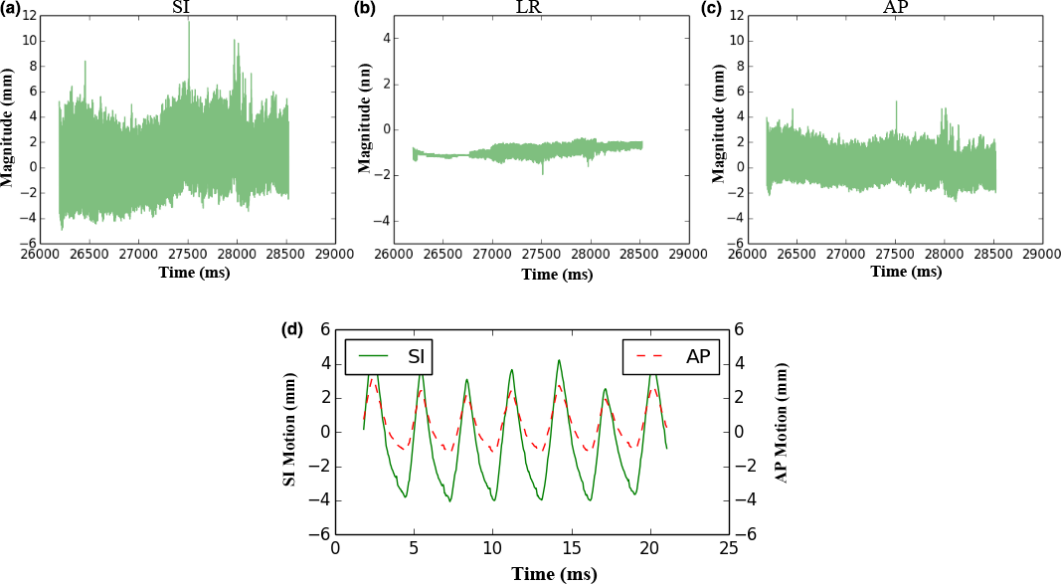
Sample of tumor motion during treatment. The most dominant motions are in the SI and AP directions (a, c). When the tumor moves to the superior position, it also tends to move to the posterior position and vice versa (d). Superior (−), Inferior (+), Anterior (+), Posterior (−).

Figure 2 shows a sample of the tumor motion during the treatment. As expected, the liver tumor made reciprocating movements in the superior–posterior direction, following the diaphragm motion. A strong correlation of tumor motion between the superior–inferior (SI) and anterior–posterior (AP) directions was observed [Fig. 2(d)]. The median (range) Pearson coefficient was 0.913 (0.745–0.994) across all fractions and patients (*P* < 0.05). No significant correlation of tumor motion was observed between the SI and LR directions.

The median (range) logfile‐based amplitudes were 11.9 (5.1–17.3) mm, 1.3 (0.4–4) mm and 3.8 (0.9–7.7) mm in the SI, LR and AP directions, respectively. The median (range) 4DCT‐based amplitudes were 9.0 (6.2–12.6) mm (SI), 0.3 (0–1) mm (LR) and 2.4 (1–5.7) mm (AP), respectively. The result of Wilcoxon signed rank test showed that the 4DCT‐based amplitude was underestimated compared with the logfile‐based amplitude (*P* = 0.024, 0.001 and 0.001 for the SI, LR and AP directions, respectively). Compared to the logfile‐based amplitude, the medians (ranges) of absolute differences from the 4DCT‐based amplitude in the SI, LR and AP directions for the 14 patients were 1.8 (1.0–8.3) mm, 0.9 (0.3–3.8) mm and 1.2 (0.1–5.1) mm, respectively.

### 3.B Intra‐and interfraction amplitude variability and baseline shift

**Figure 3:**
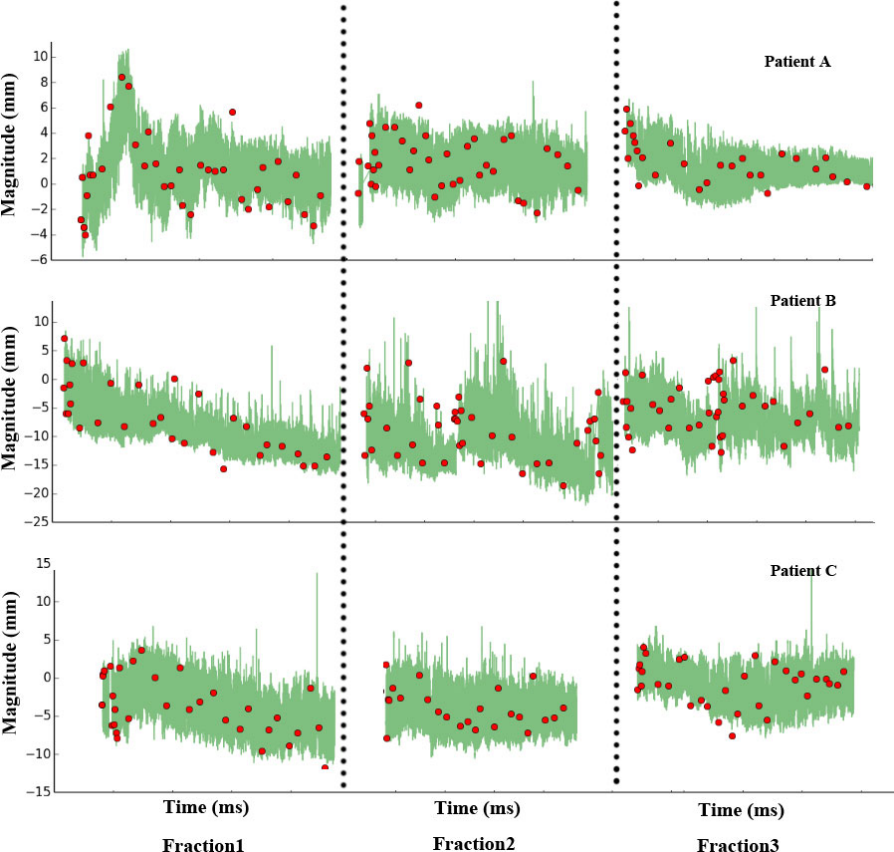
Intra‐ and interfraction motion variations in the SI direction of the first three fractions from three representative patients. The green lines are the tumor motion excursions extracted from the correlation model. The red points are the tumor positions acquired from an orthogonal X ray image system during the treatment delivery.

Figure 3 shows the tumor motion in the SI direction for the first three fractions for three representative patients. In fact, most of the patients showed clear tumor motion pattern changes during the course. The intrafraction amplitude variability included the peak‐to‐peak distances change (e.g., the 3rd fraction of patient B), and the tumor baseline position shifts (e.g., the 1st fractions of patients B and C). A considerable intrafraction amplitude variability was observed in the 2nd fraction of patient B because a dramatic peak‐to‐distance change and baseline shift occurred simultaneously. In addition, the interfraction amplitude varied significantly (patient A). The first fraction of patient A showed a dramatic tumor position variation, which may be caused by the nervousness of the patient at the beginning of the treatment. It is worth noting that the observed red points in Fig. 3 represent the tumor positions, which were obtained using the orthogonal X ray imaging system. These findings demonstrated that the tumor baseline did shift during treatment with the shifting of the external marker baseline.

**Figure 4:**
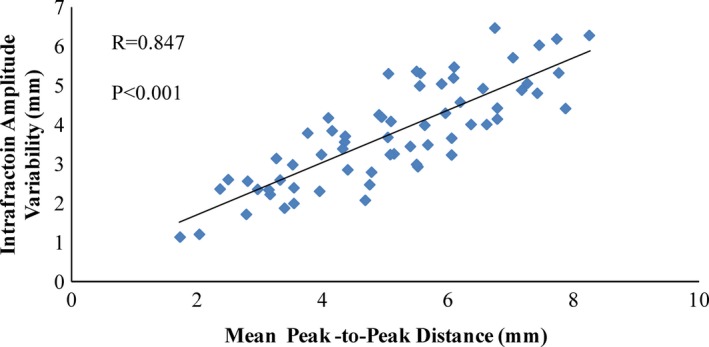
Scatter plot showing the intrafraction amplitude variability as a function of the mean peak‐to‐peak distance.

Figure 4 shows the relationship between the mean peak‐to‐peak distance and the intrafraction amplitude variability in the SI direction. The Pearson correlation coefficient was 0.847 (*P* < 0.001), indicating a strong correlation. The medians (ranges) of intrafraction amplitude variability across all patients were 4.3 (1.6–6.0) mm, 0.5 (0.2–2.2) mm and 1.5 (0.3–3.3) mm for the SI, LR and AP directions, respectively. The standard deviations of intrafraction amplitude variability among fractions, presented as the medians (ranges) of this metric across patients, were 0.5 (0.2–1.7) mm, 0.1 (0–1) mm and 0.2 (0.1–0.7) mm for the SI, LR and AP directions, respectively, which shows that the intrafraction amplitude variation has considerable stability during the treatment course. The medians (ranges) of interfraction amplitude variability across all patients were 1.7 (0.5–4.6) mm, 0.3 (0.1–3.0) mm and 0.7 (0.3–2.7) mm for the SI, LR and AP directions, respectively. An interfraction amplitude variation > 3 mm was observed in only two cases. No correlation was found between the intra‐ and interfraction amplitude variations.

**Figure 5:**
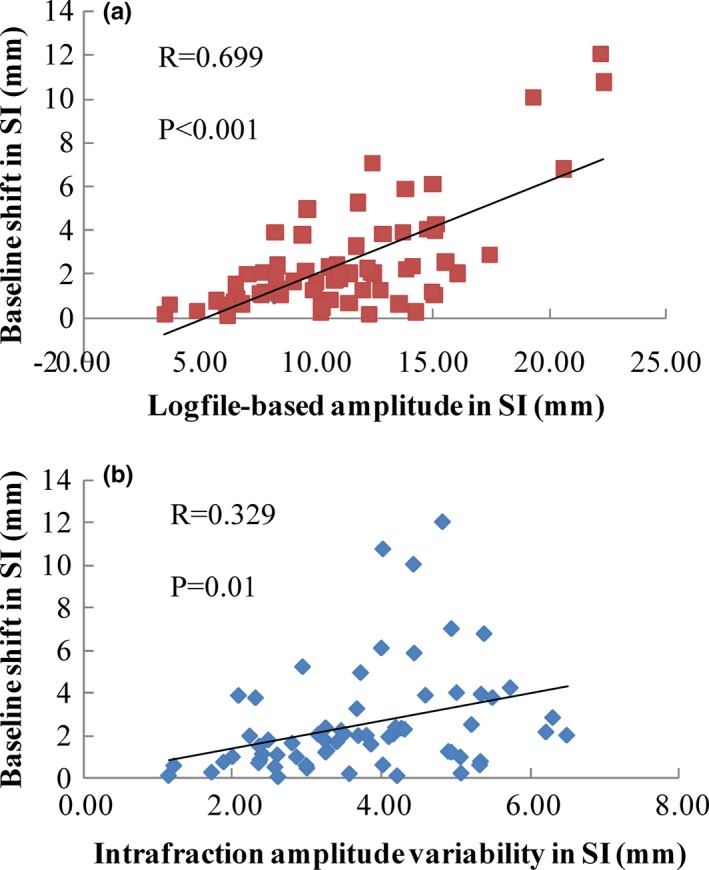
Relationship between the baseline shift and the logfile‐based amplitude in the SI direction (a) and the relationship between the baseline shift and the intrafraction amplitude variability in the SI direction (b).

The baseline shift occurs primarily toward the SI and AP directions. The medians (ranges) of baseline shift were 1.87 (0.06–12.04) mm, 0.35 (0–3.39) mm and 1 (0.02–7.21) mm for the SI, LR and AP directions, respectively, and 2.26 (0.22–14.28) mm for the 3D (square root of the sum of the squares of the distances in the SI, LR and AP directions). Pearson correlation analysis was used to determine the linear relationship between the baseline shift and logfile‐based tumor motion amplitude [Fig. 5(a)] and between the baseline shift and intrafraction amplitude variability [Fig. 5(b)] in the SI direction. A positive correlation was found for the baseline shift and logfile‐based tumor motion amplitude (*R* = 0.699, *P* < 0.001), indicating that a baseline shift is more likely to occur in those patients with large tumor motion amplitudes. However, no apparently linear trend between baseline shift and intrafraction amplitude variability was observed (*R* = 0.329, *P* = 0.01).

Table 2 presents the incidences of baseline shifts exceeding 2 mm, 3 mm and 5 mm in the three time blocks for the SI, LR, AP, and 3D directions. An apparent time trend increase in the incidence of baseline shift can be observed. Interestingly, the incidence of baseline shifts >2 mm in the SI direction for the first fraction was 35.7%, which is larger than the incidences of the 2nd and 3rd fractions, which were 21.4% and 14%, respectively.

**Table 2:** Incidence of baseline shifts exceeding 2 mm, 3 mm, and 5 mm for the SI, LR, AP, and 3D directions

	Time block 1	Time block 2	Time block 3
>2 mm			
SI	21.2%	34.5%	47.6%
LR	0.0%	3.4%	4.8%
AP	4.5%	17.2%	26.2%
3D	27.3%	50.0%	66.7%
>3 mm			
SI	6.1%	20.7%	31.0%
LR	0.0%	0.0%	2.4%
AP	0.0%	5.2%	7.1%
3D	7.6%	25.9%	38.1%
>5 mm			
SI	1.5%	13.8%	16.7%
LR	0.0%	0.0%	0.0%
AP	0.0%	1.7%	2.4%
3D	3.0%	15.5%	19.0%

## 4 DISCUSSION

The intra‐ and interfraction amplitudes clearly vary. Recently, real‐time observation of tumor movement during treatment is becoming more common,^16^, ^17^ and new apparatuses integrating this function are gradually being used by clinical facilities.^18^, ^19^ CyberKnife is the earliest commercial device for real‐time monitoring and tracking. Logfile data from the Synchrony system record the tumor motion during the treatment, and the amplitudes determined by the logfile data may be closer to reality than 4DCT or CBCT methods. Eccles et al.^20^ evaluated the liver tumor motion using cine‐MRI, and the scanning time was at least 5 min. The logfile‐based amplitudes in this study were consistent with the results reported by the above literature, which showed the amplitudes measured for long periods.

Knybel et al.^15^ analyzed the intrafraction amplitude variation in lung tumors in a large sample via CyberKnife logfiles. Most cases with intrafraction amplitude variations > 3 mm were at the area of contact with a diaphragm. The authors also noted that the intrafraction amplitude variation increased with increases in the tumor motion amplitude. Our results agree with that finding, indicating that more attention should be paid to patients with large tumor motion amplitude because these patients may have larger intrafraction amplitude variability and higher probabilities of baseline shift. Compared with the work of Knybel et al., our results show that the magnitude and incidence of baseline shift in the liver are larger than those in the lung.

Case et al.^9^ showed smaller intra‐ and interfraction amplitude variations than our results. They used diaphragm motion instead of liver tumor motion and analyzed the motion on respiratory‐correlated CBCT. Compared with the results reported by Case et al., Shimohigashi et al.^21^ decreased the liver motion amplitude using abdominal compression and significantly decreased intrafraction motion variation, but the authors reported no improvement in interfraction motion variation.

The baseline shift is an important factor for evaluating tumor motion, especially for cases with gating treatment. For gating treatment, the gating window is typically chosen such that the residual tumor motion in the gating window is less than 5 mm.^22^ When a baseline shift occurs, the tumor may move away from the gating window, resulting in a longer treatment time or an underdose at the target. Shah et al.^23^ observed the mean target position using the pre–post‐treatment CBCT of 126 patients. They reported that the incidences of mean target position shifts > 2 mm and 5 mm were 40.8% and 4.2%, respectively. A similar conclusion was made by Takao Seishin et al.,^24^ who evaluated the baseline shift by tracing the treatment couch of a real‐time tumor tracking system and noted that a longer treatment time may increase the incidence and magnitude of the baseline shift. Our results also supported their points. For gating treatment with a long treatment time, tumor position realignment before the dose delivery of each beam is an effective way to re‐determine the tumor baseline position. For those patients with larger tumor motion amplitudes, the realignment should be performed more frequently, even during the beam delivery.

Only a few studies have investigated the intrafraction rotation of implanted liver markers.^25^, ^26^ Bertholet et al.^26^ evaluated the rotations using CBCT projections and found that the population‐based means of intrafraction rotations were 3.9° (LR), 2.9° (SI) and 4.0° (AP). However, intrafraction rotations were not included in this study, which is a limitation. Moreover, based on the definition of tumor motion amplitude in this study, whether the choice of the 5% deepest inhale and exhale positions for the cutoff affects the dose of the target needs further evaluation.

## 5 CONCLUSIONS

Data from the real‐time tracking of liver tumors show tumor motion during treatment. For liver tumors, the intrafraction amplitude variations were highly correlated with the mean peak‐to‐peak distance in the SI direction. Tumor motion amplitude and treatment time may be predictive of a baseline shift.

## CONFILICT OF INTEREST

The authors declared that they have no conflicts of interest to this work.
